# An Individual Cognitive Stimulation Therapy App for People With Dementia: Development and Usability Study of Thinkability

**DOI:** 10.2196/17105

**Published:** 2020-11-16

**Authors:** Harleen Kaur Rai, Justine Schneider, Martin Orrell

**Affiliations:** 1 Division of Psychiatry and Applied Psychology Institute of Mental Health University of Nottingham Nottingham United Kingdom; 2 School of Sociology and Social Policy Law and Social Sciences Building University of Nottingham Nottingham United Kingdom

**Keywords:** dementia, cognitive stimulation therapy, eHealth, development

## Abstract

**Background:**

There is a lack of technological resources for the mental stimulation and communication of people with dementia, which can be helpful in improving cognition and quality of life. Paper-based individual cognitive stimulation therapy (iCST) for people with dementia has the potential to be adapted to a touchscreen format. This can improve accessibility and provide mental stimulation using interactive features. There is a need for a rigorous and systematic approach toward development, leading to improved suitability and implementation of the intervention, so that more people can benefit from its use.

**Objective:**

This study aims to develop and investigate the usability of Thinkability, an iCST app that can be used by people with dementia and carers on touchscreen tablets.

**Methods:**

The Medical Research Council framework for evaluating complex interventions and the Centre for eHealth Research roadmap served as frameworks for the stages of intervention and technology development. The development of the iCST app itself adopted an agile approach with elements from action research. Hence, it was developed in 3 successive *sprints* and was evaluated by relevant stakeholders at each sprint. Sprint 1 included 2 patient and public involvement (PPI) consultation meetings, sprint 2 included 1 PPI consultation meeting, and 4 focus groups and 10 individual interviews were organized in sprint 3. A feasibility trial is currently underway.

**Results:**

The findings from each sprint were used to inform the development. Sprint 1 helped to identify the relevant evidence base and explored the attitudes of people with dementia and carers toward a potential iCST app. In sprint 2, an initial prototype was evaluated in a small PPI consultation meeting. In sprint 3, feedback was gathered through a qualitative study on the quality and perceived effectiveness of the iCST app. It was well received by people with dementia and carers. A need for more updated and personalized content was highlighted.

**Conclusions:**

This study proves that an agile approach toward technology development involving all relevant stakeholders is effective in creating suitable technology. Adding to our previous knowledge of noncomputerized cognitive stimulation therapy, the release of the iCST app will make this psychosocial intervention accessible to more users worldwide.

## Introduction

### Background

The anticipated rise in the prevalence of dementia requires the development of more complex interventions to better manage the challenges of the condition [1]. To successfully implement effective interventions that are fit for purpose, there is a need for a rigorous approach toward development with the help of appropriate frameworks. The Medical Research Council (MRC) framework offers such an approach for evaluating complex interventions and describes the entire process from development to implementation [2]. Cognitive stimulation therapy (CST) is an evidence-based psychological intervention for people with dementia, which consists of mentally stimulating activities usually performed in a group setting. Previous research has shown that it can lead to improvements in cognition and quality of life (QoL) [3]. Individual cognitive stimulation therapy (iCST) is an extension of CST and is usually delivered at home by a carer. Its development using the MRC framework enabled it to be relevant and suitable for people with dementia and their carers, and iCST showed improvements in the quality of the relationship within the dyad [4,5].

The need for more complex interventions has also led to the development of a wide range of technologies, which consist of assistive devices (eg, tracking or reminder tools), touchscreen apps (eg, games, health, and fitness apps), and more [6], to better support people with dementia. However, there is a lack in the availability of apps for mental stimulation and communication targeted toward people with dementia [7,8]. Therefore, considering the evidence base behind CST and iCST, a computerized version of iCST is needed to offer iCST on a more novel and interactive platform and to increase its accessibility. For the development of novel technology, in particular, van Gemert-Pijnen et al [9] developed the Centre for eHealth Research (CeHRes) roadmap, which includes distinct development phases from contextual inquiry to operationalization. Together with the MRC framework, this enables a highly systematic approach toward the development of a computerized version of iCST to ensure that the intervention is both usable and useful for people with dementia.

### Aims and Objectives

The aim of this study is to develop an iCST app that can be used by people with dementia and carers on touchscreen tablets.

#### Objective 1

The primary objective of this study is to develop a first prototype based on an understanding of the theoretical mechanisms behind CST, iCST, and use of technology; the attitudes of people with dementia toward paper-based iCST and the iCST app; and a selection of iCST activities suitable for a touchscreen app.

#### Objective 2

The secondary objective of this study is to evaluate the first prototype in terms of clarity, suitability, and ease of use and to expand on the selection of activities for the second prototype.

#### Objective 3

The third objective of this study is to bench test the second prototype with people with dementia and carers to refine and modify the prototype and improve its usability and to develop a full list of 21 activities for the third prototype based on the findings.

## Methods

### Approach to Development

In the development of the iCST app, a research team at the University of Nottingham worked collaboratively with a software development company (Eumedianet) in Maastricht, the Netherlands. The teams adopted an agile approach during which development takes place in an iterative and dynamic manner while collaborating with all relevant stakeholders [10]. This approach is especially helpful because it encourages meaningful involvement of end users throughout development.

**Figure 1:**
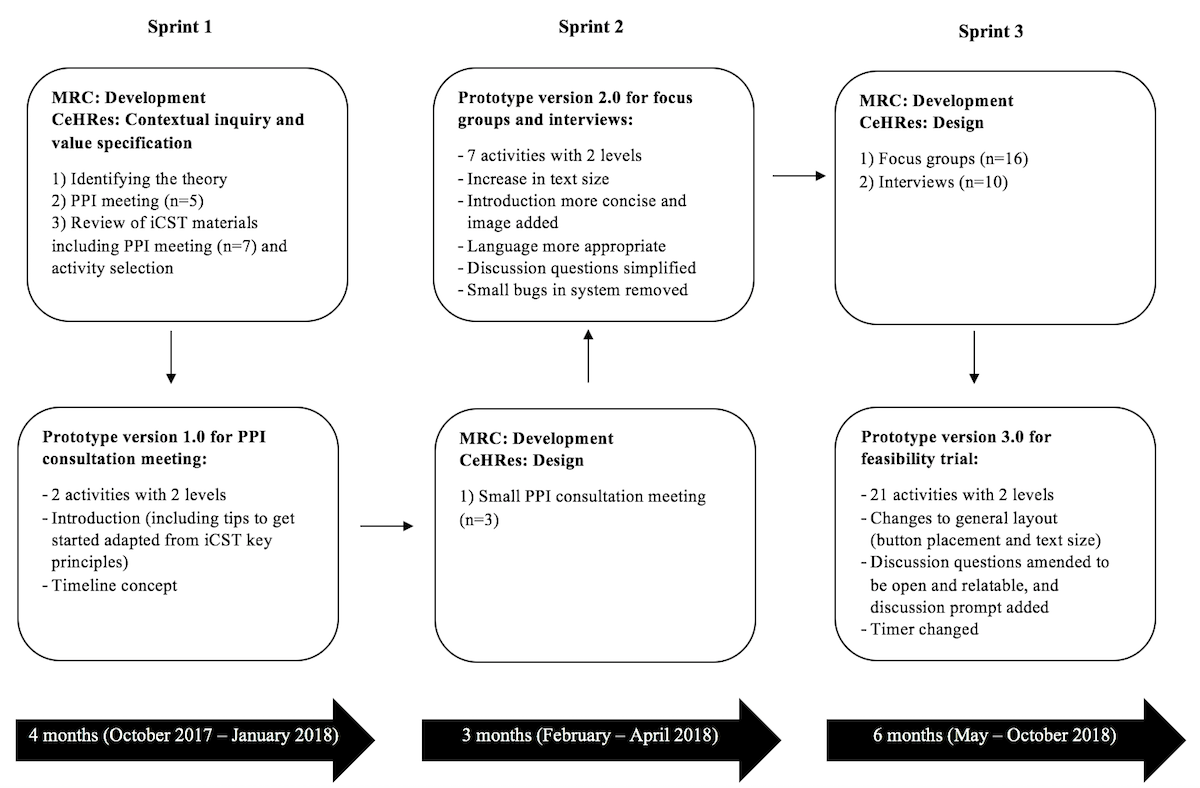
Agile development of the individual cognitive stimulation therapy app according to the Medical Research Council framework and Centre for eHealth Research roadmap. CeHRes: Centre for eHealth Research; iCST: individual cognitive stimulation therapy; MRC: Medical Research Council; PPI: patient and public involvement.

There are different types of agile development approaches (eg, Scrum, Extreme Programming, and Crystal methodologies), but they share common key principles and characteristics [10]. These include individuals and interactions over processes and tools, working software over comprehensive documentation, customer collaboration over contract negotiation, and responding to change over following a plan [11]. Scrum was chosen as the agile development method for the iCST app. This method is best suited because it focuses on efficient project management, iterative development, and feedback loops [11]. This allowed the research and software development teams, in collaboration with end users, to monitor the development of the iCST app on a regular basis and ensure that it met the necessary requirements. In Scrum, each iterative stage of development is labeled as a sprint. This led to the development of 3 prototypes over 3 sprints within the development phase of the MRC framework and the contextual inquiry, value specification, and design phases of the CeHRes roadmap (Figure 1).

The development of the iCST app also adopted elements from an action research–oriented approach. Action research seeks to use action or intervention in a cyclical research process, including the development, implementation, and evaluation of plans for practice improvement [12]. Agile development focuses on building software, whereas action research allows for a better understanding of the problem to be solved together with active participation from relevant stakeholders, including the population group. For this study, these action research elements were incorporated in the sprints to explore barriers and facilitators toward the usability and feasibility of the iCST app and identify possible solutions.

### Sprint 1: Development of Prototype Version 1.0

The first sprint consisted of 3 research activities to develop the first prototype of the iCST app: identification of the evidence base and theory behind CST and technology, a patient and public involvement (PPI) consultation meeting, and the review of existing iCST materials (including a PPI consultation meeting and activity selection). These activities reflect the recommendations of both the MRC framework and the CeHRes roadmap.

Within the development phase, the MRC framework recommends exploring the evidence base and identifying the theoretical mechanisms to better understand how an intervention can bring about change before any development work takes place [2]. Therefore, the research team reviewed the current literature on the effectiveness of CST, iCST, and the use of technology for people with dementia to better understand the mechanisms behind each component of the iCST app [13-15].

#### First PPI Consultation Meeting

To better understand the context, following the CeHRes roadmap, a PPI consultation meeting was organized with people with dementia and carers (n=5). We wanted to explore their attitudes toward a potential iCST app and to identify facilitators and barriers toward using (touchscreen) technology in general. A brief presentation was given about CST and the aims of the research project, followed by a short discussion. Topics included willingness to use an iCST app, potential benefits and limitations, and practicalities such as time investment. Notes from the meeting were communicated with the software development company.

#### Review of Paper-Based iCST Materials

The CeHRes roadmap indicates that contextual inquiry is followed by value specification, which helps to determine the most favorable solutions and features based on the values of the intended users and other stakeholders [9]. The iCST manual consists of 75 activities spread over 21 themes, leading to approximately 3 or 4 activities per theme. To better understand which features and activities should be included in the iCST app, a second PPI consultation meeting with people with dementia and carers (n=7) was organized at the Institute of Mental Health (IMH). A researcher (HR) presented a short video clip from the iCST DVD of different caregiving dyads using paper-based iCST materials. Participants were given iCST manuals and, in pairs, were asked to review the materials and discuss the qualities they liked or disliked. Participants were also asked to discuss how the iCST manual could best be adapted into a touchscreen version. A group discussion followed, and key topics included the design, content, and feasibility of a potential iCST app. The notes and contributions from the PPI meeting were fed back to the software development company.

Value specification should involve all stakeholders; therefore, both the research and software development teams also evaluated the iCST materials. Each activity was evaluated for its potential to be adapted to a touchscreen platform. Considerations included the added level of interactivity and novelty, promoting mental stimulation or the sharing of ideas and opinions, and overall enjoyment. On the basis of these considerations, all activities were first categorized per the iCST theme and the type of activity (eg, a quiz, picture game, and audio) and then ranked according to priority for development separately by the research and software development teams. After reaching a consensus in terms of priority, a small selection of activities was made, and these were developed for the iCST app prototype version 1.0.

#### Prototype Version 1.0

Following the principles of an agile approach toward development, there was a need to develop a working prototype rather than paper wireframes. This allows end users to operate a device that resembles the final product and hence to obtain more accurate feedback. In terms of design, Castilla et al [16] recommend a linear navigation over a hypertextual structure. In a linear structure, the user makes his or her way through the intervention in the order that is intended by the developer. He or she does not make decisions that reorganize the content. In a hypertextual structure, the content is rearranged based on the user’s choices, leading to nonlinear pathways [16]. The researchers assert that a linear navigation resembles an analog format of books and magazines, for instance, which are more familiar to older adults. Familiarity then further supports the learning process with the technological intervention.

**Figure 2:**
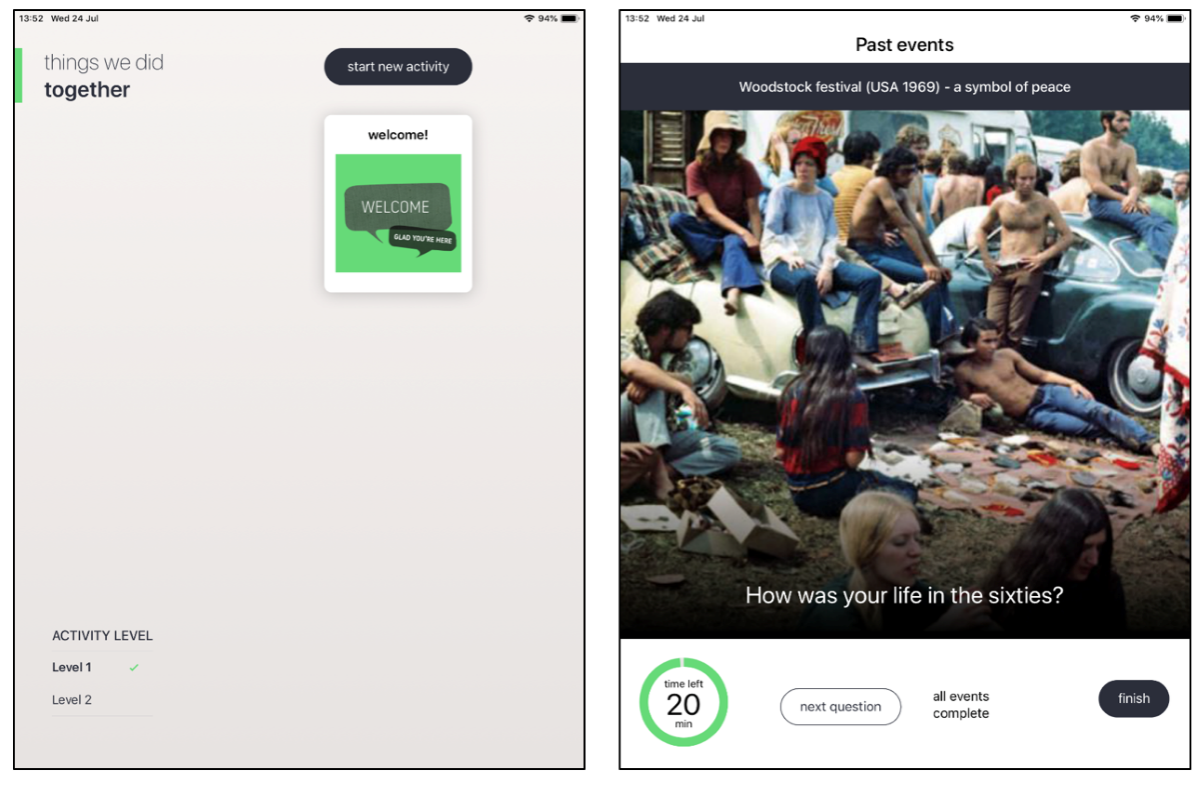
Screenshots of prototype version 1.0: home screen (left) and Past Events (right).

The first prototype consisted of several key features: a home screen, welcome or introduction, 2 activities with 2 levels of difficulty (Sounds and Past Events), and a timeline. When opening the app, users were first presented with the home screen (Figure 2). It included a welcome icon, which took the user to an introduction section. This section explained the purpose of the app and provided a few tips and aims derived from the CST and iCST principles. Furthermore, users could select a new activity on the home screen. Prototype version 1.0 included a Sounds and Past Events activity (Figure 2). A short summary preceded the actual activity to provide some instructions. Within the activity itself, there was a timer, which counted down from 20 min to keep track of the amount of time spent, and some buttons to move through the activity or finish it. Finally, each completed activity was added to the timeline on the home screen. This enabled users to keep track of their journey through the app.

### Sprint 2: Evaluation of Prototype Version 1.0

#### Third PPI Consultation Meeting

Design is the third stage of the CeHRes roadmap and is used to build prototypes that fit the user requirements. End users are invited to give feedback and test prototypes to assess whether they match their expectations in terms of system, content, and service qualities [9]. The iCST app prototype version 1.0 was taken forward in a small PPI consultation meeting with 2 people with dementia and 1 carer at the IMH. The software development team supplied the research team with a list of questions relating to clarity and overall ease of use. The main topics included the design, navigation, and content of the prototype. After an introduction and general explanation about the prototype, each participant was given a touchscreen tablet to use the prototype for 15 to 20 min. A researcher (HR) provided support and guidance in case of any difficulties and answered questions throughout the trialing period. This was followed by a group discussion of approximately 1 hour. Another researcher (JS) made observations and took detailed notes during the meeting, which were communicated with the software development team through video conferencing. Feedback from the consultation meeting was used to further expand the prototype and build version 2.0 for bench testing.

#### Prototype Version 2.0

**Figure 3:**
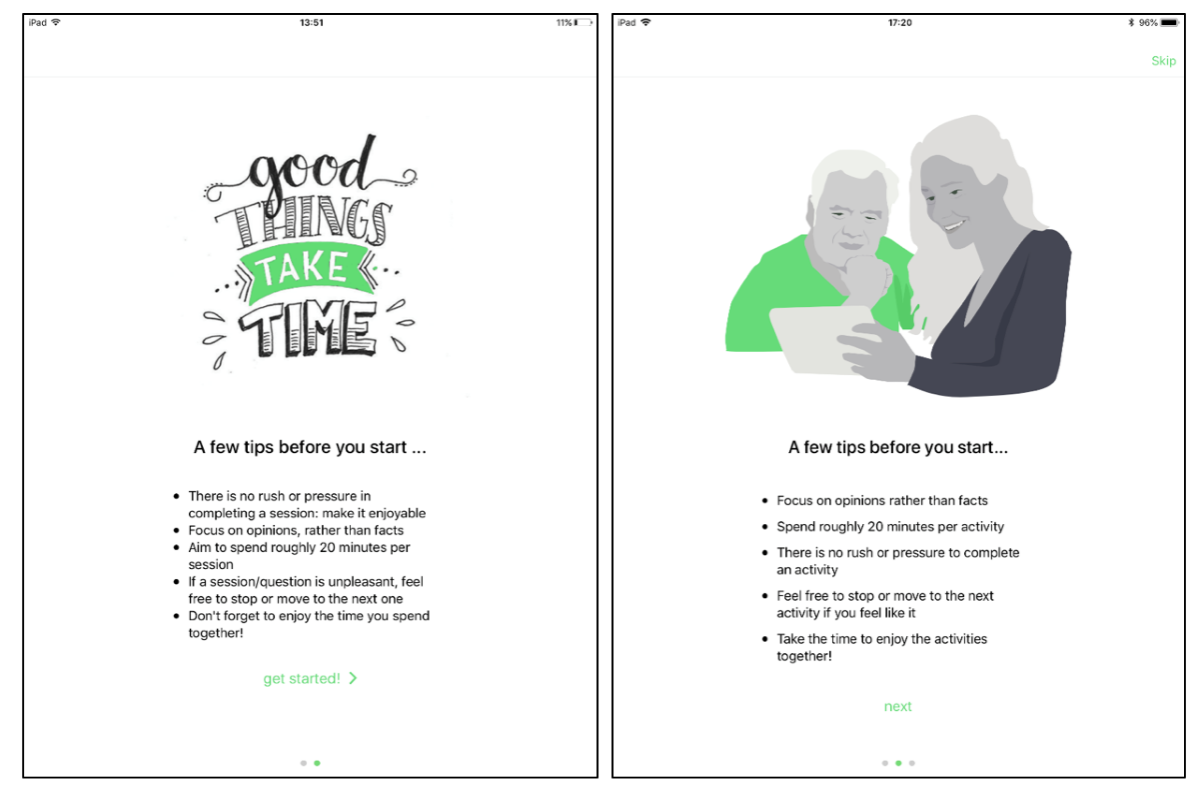
Screenshots of the introduction section of prototype version 1.0 (left) and prototype version 2.0 (right).

Following the feedback from the PPI consultation meeting, the second prototype was expanded with an additional 5 activities, making it a total of 7 (Textbox 1). These 5 activities (Garland, Hangman, Odd One Out, The Price is Right, and Useful Tips) were chosen based on the initial selection of activities during sprint 1. The aim was to have a diverse selection and therefore included several types of activities, such as a number game, categorization activity, and a video. The introduction section was simplified, and an image displaying 2 people interacting with a tablet was added (Figure 3). Finally, some bugs in the system were removed, such as incomplete captions within activities.

**Overview of activities for each prototype version.**

- Prototype version 1.0: Sounds and Past Events
- Prototype version 2.0: Sounds, Past Events, Garland, Hangman, Odd One Out, The Price is Right, and Useful Tips
- Prototype version 3.0: Sounds, Past Events, Being Creative, Spaceman, Odd One Out, The Price is Right, Useful Tips, iSpy, Trivia Quiz, Word Search, Sudoku, Globe Trotter, Sayings, My Life, Being Active, Food, Brainstorm, Arts, Old Wives’ Tales, Toys Are Us, and In Pairs

### Sprint 3: Evaluation of Prototype Version 2.0

#### Design

The evaluation of the second prototype also comprised the design phase of the CeHRes roadmap. However, focus groups and semistructured interviews were included in this sprint, according to the recommendations of the MRC framework, which enabled the gathering of more in-depth and rich qualitative data. The second prototype was presented to people with dementia and family carers for them to bench test it and modify, refine, and improve its usability. Ethical approval for the involvement of vulnerable adults with dementia and their carers was obtained through the National Health Service (NHS) Health Research Authority—Yorkshire & The Humber—Bradford Leeds Research Ethics Committee (Reference number 17/YH/0405).

#### Sample

A total of 13 people with dementia and 13 family carers participated in the focus groups and interviews (N=26). Eligibility criteria were adapted from previous iCST research [4]. Recruitment took place in primary and secondary care settings, including memory clinics, voluntary sector organizations, and support groups through the Nottinghamshire Healthcare NHS Foundation Trust.

#### Methods

In total, 4 focus groups were organized: one with people with dementia (n=4), one with family carers (n=4), and 2 mixed groups with both (n=8). In addition, 10 individual interviews were conducted with people with dementia (n=5) and family carers (n=5) in the homes of the participants. All interview participants completed an additional usability questionnaire [16]. The aim of combining these methods was to gather more diverse data. Furthermore, where focus groups allow the sharing of thoughts with others and coming to different ideas, individual interviews are more useful to allow participants to voice their opinions without the potential group influences. Discussion guides were developed by the research team and included a range of topics, such as the layout and content of the prototype, using it as a dyad, and any practicalities and general points related to the prototype (Multimedia Appendix 1).

Participants were asked to trial the app in pairs for 10 to 15 min before the discussion, while 2 researchers gathered observational data as per the recommendations of the software development team. Guidance from the researchers was kept at a minimum to investigate whether the app was intuitive. However, the researchers provided support when participants had any questions. The feedback and contributions from this qualitative study supported the development of the iCST prototype version 3.0.

#### Analysis

The data from the focus groups and interviews were audio recorded and transcribed by the research team. The data were coded by 2 researchers using inductive thematic analysis to identify the key themes within the data [17]. The findings were further supported by observational data.

#### Prototype Version 3.0

**Figure 4:**
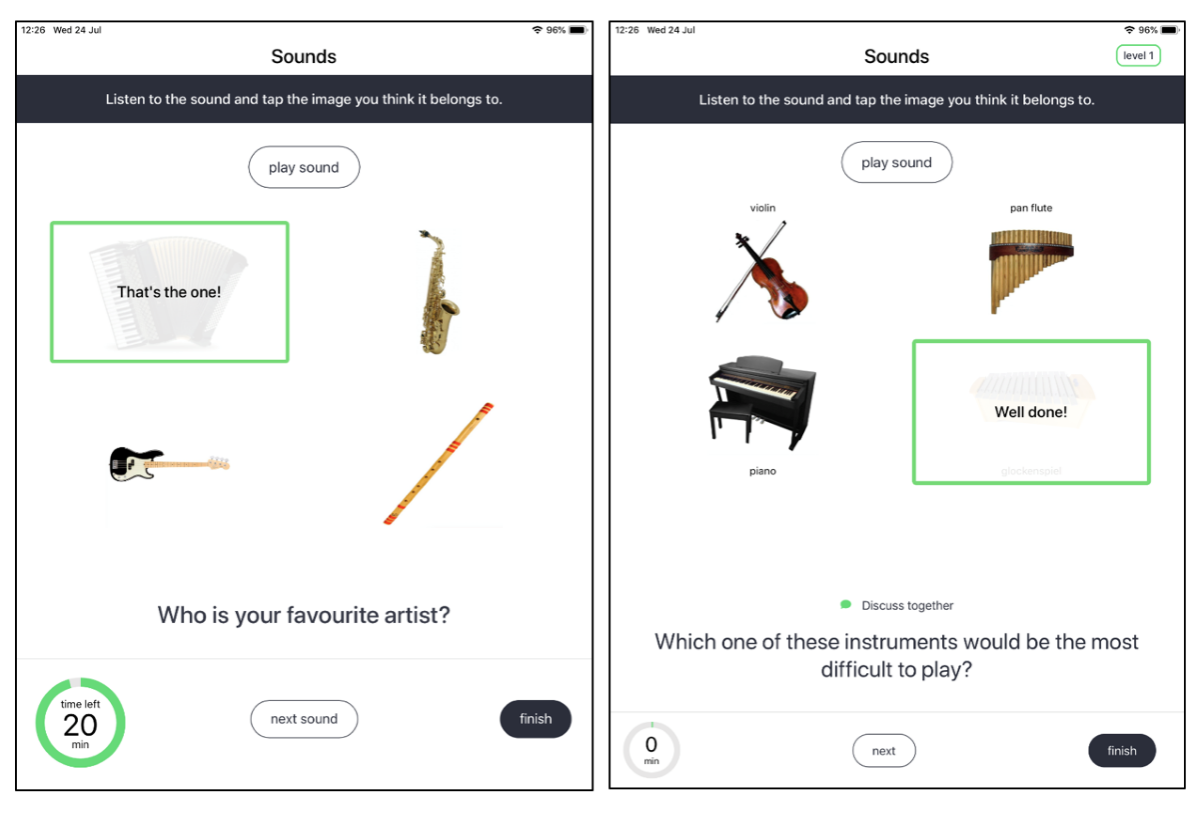
Screenshots of the Sounds activity in prototype version 2.0 (left) and prototype version 3.0 (right).

On the basis of these findings, the third prototype was expanded with a full range of 21 activities to be taken forward in a feasibility trial (Textbox 1). As participants were happy with the diversity of the activities in prototype version 2.0, the teams decided to continue building the remainder of the activities that were selected in sprint 1. Some suggestions for new activities given by the participants were incorporated into the prototype version 3.0, such as a word search and a quiz. The majority of the improvements were related to the design of the app and activities (Figure 4). For instance, some participants felt rushed while doing an activity because of the timer counting down the amount of minutes. Therefore, the timer was changed to count up to 20 min, with participants being able to spend more time on it if they wanted to (Figure 4). Furthermore, the level of the activity was added to the top-right corner. The activity Hangman was changed to Spaceman, as the initial icon image included a noose, which was too negative. The language was deemed appropriate and free of jargon; however, more changes to the discussion questions were necessary. Hence, the questions were written to be more open and relatable. In addition, some participants suggested adding a little prompt above the question saying *discuss* to clarify the purpose of the questions. Finally, some more context was provided to the Garland activity to clarify that it is an activity that can be done without the tablet.

## Results

### Sprint 1: Development of Prototype Version 1.0

#### Identifying the Evidence Base and Theory Behind CST and Technology

A recent book published by CST research members served as the main resource for key evidence relating to CST and iCST. Both group CST and iCST were developed following the MRC framework, which helped to create a strong foundation for both interventions [18]. For group CST, a large-scale randomized controlled trial (RCT) demonstrated positive effects on the cognitive functioning and QoL of people with dementia, which were further supported by qualitative findings [3,19]. CST is multifaceted, and its key principles reinforcing mental stimulation, use of reminiscence, and enjoyment, contribute to its effectiveness. Evidence suggests that stimulating activities targeting certain neuropsychological domains, similar to those in CST, can improve cognition [20]. Furthermore, CST provides a social context for its participants, and previous research suggests that social interaction between 2 or more individuals can improve cognition [21]. The effects of CST on QoL may be explained through the mediating role of improvements in cognitive functioning [13].

The large-scale RCT with iCST did not have positive effects on cognition and QoL for people with dementia, which could perhaps be because of a low completion rate of the planned activities. Instead, researchers found improvements in the quality of the caregiving relationship between the person with dementia and the carer [4]. Although iCST is as multifaceted as group CST, the trial results may be the result of a lack of adherence to the intervention. Alternatively, the lack of a social setting in iCST may have contributed to the results. Researchers emphasize the need for more research, particularly with experimenting with computerized platforms for providing iCST. Novel cognitive stimulating activities that promote the learning of a new skill can benefit the cognitive functioning of older people [14]. For example, Chan et al [22] found that training healthy older adults to use iPads (Apple Inc) could lead to benefits in episodic memory and processing speeds. Increased processing speeds are especially beneficial, as they are associated with improved execution of various technological tasks [23].

Computerized cognitive interventions for people with dementia are becoming increasingly widespread. Garcia-Casal et al [15] concluded that computerized cognitive interventions led to significant improvements in cognition, depression, and anxiety among people with dementia. Therefore, computerized cognitive interventions may have even more of an impact on cognition than noncomputerized cognitive interventions such as group CST [15]. However, these interventions can vary greatly; therefore, there is a need for more research, with computerized cognitive stimulation in particular, to better understand the effects. The platform on which an intervention is offered is also important, for instance, a touchscreen device or a computer. There is considerable evidence suggesting that touchscreen tablets are highly intuitive for older people with dementia [24,25]. Moreover, Tyack and Camic [26] found that touchscreen interventions, which are simple, intuitive, aesthetically pleasant, and error free, can lead to several benefits for people with dementia, including mood, mental health, and social relationships. The intervention should include slightly challenging content so that the user is invited to apply more complex cognitive skills rather than simpler ones [26].

Despite the current evidence and available technological resources, there is still a need for more technologies that provide people with dementia with independent activities for mental stimulation, enjoyment, and a meaningful way to spend time [7,8]. An iCST app with appropriate content and design is well placed to contribute to the current lack of technologies for mental stimulation and enjoyment.

#### First PPI Consultation Meeting

Participants were particularly enthusiastic about CST and said they would welcome it in any format, whether this was computerized or paper based. They said that researchers would have to keep a few things in mind when developing a computerized version of CST, namely, that there would be a need for personalization according to the person’s background and a diverse selection of activities. Some participants mentioned the need for a facilitator to provide support for the activities. This could be an informal or a paid carer. Being able to keep track of which activities were done and when was also considered to be a useful feature.

Attitudes toward technology were diverse, with some more willing to use technology than others. A person with dementia mentioned that she would not want to be pushed to use technology, which might happen through the involvement of a carer. However, there was consensus among the group that people with dementia need to be empowered and to be made aware of how to handle technology. An example was given on how some people with dementia with a lack of experience with technology may think a piece of technology could break easily by pressing the wrong button. People would need an explanation on how to use the actual technology before using any kind of app on it. Finally, for technology to be useful for people with dementia, it should be free of jargon and difficult terminology as much as possible.

#### Review of Paper-Based iCST Materials (PPI Consultation and Activity Selection)

All participants liked the iCST manual in terms of content and usefulness, and the comments for improvements were mostly related to practicalities and some layout issues for a potential iCST app. For instance, participants agreed that there was too much text on one page and that this would have to be minimized significantly for an app. Keeping with this, although the content was perceived to be useful, participants felt that there were too many activities and that researchers would need to consider which activities could be better for online adaptation onto an app than others.

In terms of feasibility, flexibility was considered to be one of the most important needs for an iCST app. The amount of time needed to complete one activity, and to make their way through the entire app would differ between people with dementia. Therefore, people should be able to use it according to their own pace and decide how much time they would like to spend on the app per day. One person with dementia was keen on using the app for any amount of recommended time as long as it could benefit her. Participants also emphasized some challenges. For example, it might be difficult for some users to maintain concentration for a certain period. In addition, their physical condition might prevent them from using the app (eg, pains).

After the PPI consultation meeting, the research and software development teams reviewed each iCST activity in detail. All 75 activities were ranked according to the type of activity and its potential for adaptation to a touchscreen platform. Following the advice from the PPI group members, the researchers decided to reduce the number of iCST activities from 75 to 21 for the initial iCST app. This encompasses one activity per theme.

### Sprint 2: Evaluation of Prototype Version 1.0

The design was evaluated positively with a minor suggestion to increase the size of the text. The use of colors was deemed appropriate as well. One example came from a person with dementia who did not seem to have any problems with the color scheme despite being color blind. The navigation was intuitive, as participants were able to move through different parts of the prototype with little difficulty. However, the purpose of the timeline was not clear and needed additional explanation from the researcher.

In terms of content, the participants were positive about the type of activities and found them relevant and enjoyable. To encourage discussion based on the questions, it was suggested to simplify the questions by directing them to the person with dementia rather than a general question. Participants also looked at the introduction section and suggested adding an image of a person with dementia and a carer using the app together to clarify how it is meant to be used (Figure 3). Some changes were suggested to the language to make it more suitable. Suggestions included shortening the sentences and improving the overall sentence structure. Some words were discussed in more detail, for example, using *finish activity* rather than *stop activity*. Finally, participants were keen on seeing more levels included in the future.

All but few suggestions were included in the next iteration of the prototype. For instance, there was a need to add buttons on the screen to adjust for the sounds and brightness; however, there was a potential that this would have made the interface more crowded and therefore less intuitive. We decided to further investigate this in the next sprint.

### Sprint 3: Evaluation of Prototype Version 2.0

In total, 13 people with dementia and 13 carers participated in the qualitative study. The majority of the people with dementia were male (n=8), and the majority of carers were female (n=9). The mean age of people with dementia was 74.23 (SD 6.06) years, and the mean age of the cares was 69.15 (SD 9.32) years. Most people with dementia (n=10) and all carers (n=13) had some experience with using technology. A total of 4 main themes emerged from the analysis: approaches to technology, quality of the iCST app, perceived benefits of the iCST app, and involvement of a relative or friend.

The majority of the participants were enthusiastic about the app and found it to be useful. Some participants also appreciated the novelty of the intervention:

I think it’s nice to have something different every so often. Yeah it’s something different because it’s not something that crept up before shall I say.

Person with dementia, interview 5

Observations indicated that the app was intuitive for most participants, which was confirmed through discussions and usability questionnaires. There were some cases where researchers needed to provide some assistance, and although the navigation was generally considered to be appropriate, there was a need for better signposting and clearer button placement:

For navigation purposes I found it difficult if I wanted to go back and start another one. [...] I thought that one [button] could be bigger...or more obvious.

Carer, interview 2

Participants noted that the images and text could both be slightly bigger, but overall they were rated well in terms of clarity:

I understand what each one is showing and that’s all that’s necessary for it to do. So long as the image is clear I don’t see a problem, and generally speaking they are clear.

Person with dementia, interview 7

Finally, there was no general consensus on the color scheme, with some participants opting for the inclusion of more colors and others preferring the current scheme with fewer colors to avoid distractions.

Table 1 describes the usability and acceptability of the iCST app according to people with dementia (n=5) and carers (n=5) who participated in the individual interviews. Item 5 includes 1 missing response from a person with dementia. Overall, the iCST app was rated well in multiple areas, namely, its ease of use, usefulness, and suitability of the letter or button size for both people with dementia and carers, suggesting that the overall design was appropriate. Although most participants indicated that they knew what to do at any given time, carers felt more confident while using the app than people with dementia and were also more willing to use it frequently. This suggests that the navigation of the iCST app might not be as intuitive for people with dementia as it is for carers.

**Table 1:** Results from the usability questionnaire with the individual cognitive stimulation therapy app prototype version 2.0.

Questionnaire item	Person with dementia (n=5), individual responses, n (%)		Carer (n=5), individual responses, n (%)	
	Positive	Do not know or negative	Positive	Do not know or negative
Ease of use	5 (100)	0 (0)	5 (100)	0 (0)
Usefulness	5 (100)	0 (0)	5 (100)	0 (0)
Knew what to do at any time	4 (80)	1 (20)	5 (100)	0 (0)
Felt confident while using the app	3 (60)	2 (40)	5 (100)	0 (0)
Feeling while using the app	3 (60)	1 (20)	5 (100)	0 (0)
Suitability letter or button size	5 (100)	0 (0)	4 (80)	1 (20)
Willingness to use the app often	3 (60)	2 (40)	5 (100)	0 (0)

The majority of the suggestions for improvements and additions to the app were made, and these were taken forward in the next iteration for the feasibility trial, which has been registered on the Clinical Trials website (registration number NCT0328277).

## Discussion

### Principal Findings

This is the first study to create an interactive, touchscreen iCST app for people with dementia and carers based on the principles of CST and iCST. The systematic approach to development included an agile methodology, principles from action research, and guidance from the MRC framework and CeHRes roadmap [2,9]. To the best of our knowledge, this is also the first study to combine these elements in the development of a technology-based intervention for people with dementia and carers. Within this approach lie the strengths of this study. For instance, the agile methodology helped to create and regularly review prototypes of the app in an iterative manner. This was necessary not only to monitor the overall progress and direction of development but also to find and resolve any faults on a continuous basis. Both the MRC framework and the CeHRes roadmap helped to better define the development process and determine which research activities were necessary at each stage. In lieu of this, there were various diverse research activities ranging from PPI consultations to a qualitative study and questionnaires, leading to the collection of in-depth and rich data. Finally, the involvement of end users at each stage, as per the principles of action research, was beneficial as their consistent and useful feedback helped to refine the iCST app prototypes.

The MRC framework recommends using the best available evidence and appropriate theory in the development of a new intervention [2]. Sprint 1 supported this and pinpointed several mechanisms behind the use of CST and technology, such as mental stimulation of neuropsychological domains, providing a social context, and learning a new skill [14,20,21]. The combination of these elements might be able to demonstrate how an iCST app can benefit cognition compared with paper-based iCST, which did not find such benefits for the person with dementia. An iCST app would also allow for improved monitoring of adherence to the intervention through analytical data, which was a challenge in previous iCST research [4]. This would provide further insights into the potential benefits of an iCST-based approach. Furthermore, as part of contextual inquiry, researchers explored the attitudes of people with dementia and carers toward technology. Attitudes were varied, but most participants were willing to use technology as part of their daily lives. The most important conclusion was that people with dementia would need individually tailored support on how to use the technology. This is in accordance with previous research showing that technology can be helpful for people with dementia, but some might require continuous support and education to maximize the benefits [27]. This support could be provided through the involvement of skilled practitioners or informal carers. In 2018, it was estimated that 42% of people aged ≥65 years in the United Kingdom use a touchscreen tablet compared with any other device to access the internet, making it the most popular choice among the age group [28]. However, there is a need to increase education and awareness regarding the use of technology, such as touchscreen tablets to support the empowerment of people with dementia, which was another prerequisite mentioned in the first PPI consultation meeting. However, some people may be unable to access technology and thus the iCST app, and the availability of other CST resources such as group CST and paper-based iCST will help them access a form of CST.

Through value specification, people with dementia and carers were asked to identify their most important needs for an iCST app. The identification of needs is a common process in the development of technology, as it helps to define and prioritize user requirements. In PPI consultation meetings, people with dementia and carers stressed the need to minimize the current paper-based iCST content, flexibility in using the iCST app, and a diverse range of activities to appeal to personal interests. These requirements are supported by Tyack and Camic [26], who found that touchscreen interventions should be tailored where possible in terms of content but should also include a simple and intuitive interface. This can facilitate the uptake of the intervention. Minimizing the iCST content would make the interface less crowded and easier to use. Value specification was continued by both the research and the software development teams to assess which paper-based iCST activities should be taken forward in the iCST app. On the basis of priority ranking of each team, a small first prototype was developed.

In sprint 2, the development moved toward the design aspect of the CeHRes roadmap during which the first version of the digital health intervention is communicated with end users to collect feedback. It is recommended to initially present a prototype that does not fully resemble the final product but does include the essential features and then build on successive prototypes [9]. Therefore, the iCST app prototype version 1.0 only included 2 activities in addition to the main features (eg, the timeline). Participants in a PPI consultation meeting rated the format of the iCST app prototype version 1.0 positively, particularly the design was deemed appropriate. However, there was a need to simplify the content. For instance, the introduction contained some jargon, and the discussion questions needed to be clearer. This feedback informed the expansion of the next prototype, which better resembled the final product.

Sprint 3 was an extension of the design stage of the CeHRes roadmap and included more formal usability testing through focus groups, interviews, and questionnaires. These activities are also recommended by the MRC framework to assess the acceptability of the intervention [2]. The iCST app prototype version 2.0 was evaluated positively, and participants gave some suggestions for improving the design, including an increase in the size of the text and images.

### Limitations

Working agile requires a quick turnaround for prototypes in terms of development and evaluation. The latter proved more challenging, as research activities with end users require a sufficient amount of time for recruitment and organization. For instance, the lack of time caused the evaluation of prototype version 1.0 in sprint 2 to be less in depth. An additional challenge in recruitment was that the PPI consultation meeting contained a small sample size, potentially leading to insufficient data and feedback. To better cope with these challenges and add more value to development, it is recommended to involve 1 or 2 people with dementia as co-researchers throughout the development process to receive consistent feedback.

### Future Research

Future studies involving new technology-based interventions for people with dementia will need to establish a strong collaboration with researchers, software developers, and end users from the beginning stages of development. Furthermore, new interventions and their development will need to be supported by appropriate frameworks and methodologies. These recommendations will help to create an intervention, which is fit for purpose and has better potential to be successfully implemented in practice.

This study did not include the last development phases of piloting, evaluation, and implementation. Therefore, the next iCST app prototype (version 3.0) will now be taken forward in a feasibility trial to better understand its acceptability, usefulness, and any potential signs of the effectiveness of the iCST app in daily life. Additional future activities could support the promotion and dissemination of the iCST app. Some examples include collaborations with international Alzheimer associations, visits to local memory cafes and dementia support groups in the community, and media exposure through the newspaper or radio.

### Conclusions

This study demonstrates that an agile approach toward technology development involving all relevant stakeholders can be effective in creating suitable technology for people with dementia. This process can be further supported by using appropriate frameworks to better understand the development process and determine the necessary research activities. Furthermore, this study demonstrated that there is an interest and willingness to use an iCST app among people with dementia and carers. Therefore, these results have been added to our previous knowledge of paper-based CST, and a commercial release of the iCST app will strengthen CST’s current international impact by making it more accessible to users around the world.

## Appendix

Multimedia Appendix 1 Interview discussion guide for sprint 3.

## Abbreviations

CeHRes: Centre for eHealth Research
CST: cognitive stimulation therapy
iCST: individual cognitive stimulation therapy
IMH: Institute of Mental Health
MRC: Medical Research Council
NHS: National Health Service
PPI: patient and public involvement
QoL: quality of life
RCT: randomized controlled trial
