# Supplementary material for: An Individual Cognitive Stimulation Therapy App for People With Dementia: Development and Usability Study of Thinkability
Source: JMIR Aging. 2020 Nov 16;3(2):e17105. doi: 10.2196/17105 (PMC7704283; doi:10.2196/17105)
Supplement: Multimedia Appendix 1 [file aging_v3i2e17105_app1.docx]

**Interview Guide – iCST app – Sprint 3**

1. Introductions

- Short introduction and ask the participant to tell how he/she feels today.

(5 min)

2. iCST application: after the try-out session, we will ask the following questions.

iCST app lay-out:

- What do you think about the size and font of the text?
- What do you think about the images e.g. size?
- Is the lay-out clear?
- What do you think about the use of colours?

(5 min)

iCST app content:

- What do you think about the content of the current sessions? E.g. diversity, relevance, enjoyment?
- Are concepts and terms explained well?
- Is the language used appropriate and easy to understand?
- Any mistakes in spelling / grammar?
- Are there any other features you think could benefit the iCST app?

(15 min)

3. Using the iCST app together:

- Are there any activities you do together?
- Would you consider using this application at home with one of your relatives or a close friend?
- If yes, what do you think about using this application together with the person you are caring for/you care?
- What are some of the benefits or disadvantages you can think of while using the iCST app together?

(15 min)

4. Practical issues related to using the iCST app

- Would you have the time to spend 1.5 hours on the iCST app a week at home? Would you like to as well?
- Can you foresee any practical difficulties/challenges you might face if you were to use/deliver (for the carer) this application?
- What kind of support do you think you might need if you were to use/deliver (for the carer) this application?

(10 min)

5. General points about the iCST app:

- Overall, what do you like / dislike about the iCST app?
- Do you think it would be easy to use? Why or why not?
- Compared to a paper-based version, what do you think could be the additional benefits of computerised iCST?
- Are there any other comments you would like to make?

(10 min)
